# The lung function score and its components as predictors of overall survival and chronic graft-vs-host disease after allogeneic stem cell transplantation

**DOI:** 10.3325/cmj.2016.57.16

**Published:** 2016-02

**Authors:** Diana Ditz, Robert Rabanus, Christian Schulz, Daniel Wolff, Barbara Holler, Ernst Holler, Gerhard Carl Hildebrandt

**Affiliations:** 1 Department of Internal Medicine III, University of Regensburg Medical Center, Regensburg, Germany; 2 Department of Internal Medicine II, University of Regensburg Medical Center, Regensburg, Germany; 3 Division of Hematology and Blood and Marrow Transplantation, Markey Cancer Center, University of Kentucky, Lexington, KY, USA

## Abstract

**Aim:**

To retrospectively assess if the modified lung function score (LFS) and/or its components, forced expiratory volume within the first second (FEV_1_) and diffusion capacity for carbon monoxide corrected for hemoglobin level (cDLCO), predict overall survival (OS) and chronic graft-vs-host-disease (cGvHD).

**Methods:**

We evaluated 241 patients receiving allogeneic hematopoietic stem cell transplantation (allo-HSCT) at the University of Regensburg Transplant Center between June 1998 and July 2005 in relation to their LFS, FEV_1_ and cDLCO, before and after HSCT.

**Results:**

Decreased OS after allo-HSCT was related to decreased pre-transplantation values of FEV_1_<60% (*P* = 0.040), cDLCO<50% of predicted value (*P* = 0.025), and LFS≥III (*P* = 0.037). It was also related to decreased FEV_1_ at 3 and 12 months after HSCT (*P* < 0.001 and *P* = 0.001, respectively) and increased LFS at 3 and 12 months after HSCT (*P* = 0.028 and *P* = 0.002, respectively), but not to changes of cDLCO. A higher incidence of cGvHD was related to decreased FEV_1_ at 6, 12, and 18 months (*P* = 0.069, *P* = 0.054, and *P* = 0.009, respectively) and increased LFS at 12 months (*P* = 0.002), but not to changes in cDLCO.

**Conclusions:**

OS was related to both LFS and FEV_1_, but cGvHD had a stronger relation to FEV_1_ than to cDLCO or LFS. FEV_1_ alone offered more information on the outcome after allo-HSCT than LFS or cDLCO, suggesting limited value of LFS for the patients’ assessment after allo-HSCT.

Pulmonary complications significantly contribute to late-onset morbidity and mortality after allogeneic hematopoietic stem cell transplantation (allo-HSCT). Patients with pulmonary dysfunction surviving longer than 2 years had a 15.1-fold increased risk of late mortality than the general population (1). Late onset non-infectious pulmonary complications can present in different forms, such as restrictive changes on pulmonary function testing (PFT) only, late interstitial pneumonitis (IP), cryptogenic organizing pneumonia (COP), airflow obstruction detected by PFT only, or bronchiolitis obliterans (BO)/bronchiolitis obliterans syndrome (BOS). Both, restrictive or obstructive changes can occur isolated or in combination (2-5). Although the only currently accepted form of chronic graft-vs-host-disease (cGvHD) of the lung is BO/BOS, it seems that all forms can occur associated with cGvHD and, although not pathophysiologically fully understood, may reflect potential overlapping forms or different phenotypes of pulmonary cGvHD (2-11). BO/BOS is presumably the most detrimental form characterized by frequent non-responsiveness to treatment, progressive clinical course, and irreversibility, all of which contributes to its high morbidity and mortality (12,13).

cGvHD is a major complication in long-term survivors after allo-HSCT (1,14,15), with a 6-year incidence of up to 61% in patients receiving peripheral blood stem cells (PBSC) (15) and with relevant impact on the quality of life in many patients (16-18).

The lung function score (LFS) combines the forced expiratory volume in the first second (FEV_1_) and the diffusion capacity of the lung for carbon monoxide corrected for hemoglobin level (cDLCO) in an equally distributed manner. The LFS was first proposed by Parimon et al (19) as an approach to correlate PFT results prior to allo-HSCT with the clinical outcome. Later it was modified into more precise subcategories by the National Institute of Health (NIH) Consensus Development Project on the criteria for clinical trials in cGvHD (Table 1) and suggested as a score to quantify pulmonary cGvHD and evaluate the effect of cGvHD treatment (20). In our clinical practice, we have seen cDLCO decreasing already after induction of treatment and remaining low for several months after allo-HSCT without obvious impact on the outcome. Therefore the aim of this study was to evaluate the association of pre- and post-HSCT LFS, defined according to the NIH consensus development project definition (20), and the LFS constituting parameters cDLCO and FEV_1_ individually, with overall survival (OS) and development of cGvHD after allo-HSCT.

**Table 1:** Lung function score (LFS) according to Pavletic et al (20)*

FEV_1_ in % of predicted	cDLCO in % of predicted	Score		Σ score (FEV_1_ + cDLCO)	LFS
>80	>80	1	Normal	2	I
70-79	70-79	2	Mild decrease	3 - 5	II
60-69	60-69	3	Moderate decrease	6 - 9	III
50-59	50-59	4	Severe decrease	10 - 12	IV
40-49	40-49	5			
<40	<40	6			

*FEV_1_ – forced expiratory volume in the first second; cDLCO –.diffusion capacity of the lung for carbon monoxide corrected for hemoglobin level.

## Patient characteristics and methods

### Patient characteristics

This retrospective single-center study included 241 out of 247 adult patients of Caucasian origin who received allo-HSCT at the University of Regensburg Medical Center, Regensburg, Germany between June 1998 and July 2005; 6 patients were excluded due to missing data on pulmonary function before allo-HSCT. The median follow-up was 711 days (range, 22-3091 days) and the last day of recording data was March 31, 2007. Mean age was 44.5 years, 39% of patients were female and 69% male, 48% had a related and 52% an unrelated donor, 46% of patients had Eastern Cooperative Oncology Group (ECOG) index 0, 46% had ECOG index 1, and only 2% had ECOG index 2.

Prior to transplant, patients gave informed consent on the use of patient- and treatment-related information for retrospective analyses and publication. Standard myeloablative conditioning regimens consisted mainly of 8-12 Gy fractionated total body irradiation followed by high dose cyclophosphamide +/− fludarabine or classic busulfan/cyclophosphamide, whereas reduced intensity conditioning (RIC) consisted mainly of the FBM (fludarabine/BCNU/melphalan) regimen (21). T-cell depletion for unrelated donor HSCT was performed by serotherapy with antithymocyte globulin (ATG) in 147 patients, with alemtuzumab in 4 patients, and with ex vivo selection of donor CD34+ cells in 24 patients. The severity of acute GvHD was graded from 0 to 4 using the Glucksberg scale (22). cGVHD was classified into no, limited, and extensive disease according to Shulman et al (23) and grouped by the presence or absence of cGvHD (Table 2).

**Table 2:** Patient characteristics regarding allo-HSCT and disease. Stage of disease is defined as 1 for first complete remission of acute leukemia or non-Hodgkin lymphoma or chronic phase of chronic myeloid leukemia)*

Characteristics	n = 241	(%)
Sex		
female	95	(39)
male	146	(61)
Disease		
acute leukemia, myelodysplastic syndrome	127	(53)
chronic myeloid leukemia	25	(10)
Hodgkin´s disease	6	(3)
non-Hodgkin lymphoma	45	(19)
multiple myeloma	18	(7)
myeloproliferative disease	10	(4)
other	10	(4)
Stage of disease at allogeneic hematopoietic stem cell transplantation (allo-HSCT)		
1	36	(15)
>1	204	(85)
NA	1	(0)
Therapeutic radiation		
yes	22	(9)
no	219	(91)
Smoker		
yes	86	(36)
no	135	(56)
NA	20	(8)
Pulmonary disease before allo-HSCT		
yes	57	(24)
no	147	(61)
NA	37	(15)
Donor type		
matched related donor	115	(48)
matched unrelated donor	126	(52)
Treatment related mortality:		
yes	66	(27)
no	175	(73)
Eastern Cooperative Oncology Group index before allo-HSCT		
0	111	(46)
1	111	(46)
2	5	(2)
Cytomegaly virus reactivation risk		
negative/negative	93	(39)
donor negative/recipient positive	41	(17)
donor positive/recipient negative	39	(16)
positive/positive	66	(27)
NA	2	(1)
Conditioning regimen		
reduced intensity conditioning	126	(52)
myeloablative	115	(48)
T-cell depletion (with antithymocyte globulin, Campath or selected peripheral blood stem cells)		
yes	175	(73)
no	66	(27)
Total body irradiation		
yes	120	(50)
no	121	(50)
Busulfan		
yes	9	(4)
no	232	(96)
Stem cell source		
peripheral blood stem cells	193	(80)
bone marrow	48	(20)
Acute graft-vs-host-disease (GvHD)		
no or stage 1	110	(46)
stage >1	120	(50)
NA	11	(4)
Chronic GvHD		
no	132	(55)
yes	109	(45)

*Mean patient age: 44.5 years (17-68 years); mean donor age: 40.2 years (13-67 years).

PFT was scheduled before allo-HSCT and 3, 6, 9, and 12 months after transplant. Thereafter, patients were supposed to return to the center at 6-month intervals for follow-up or at shorter intervals if clinical complications were present. PFT was performed in our center according to the guidelines of the European Respiratory Society using the MasterScreen Body (Viasys Health Care, Würzburg, Germany) including spirometry, body plethysmography, and diffusion capacity measurements using the single breath method. The data were digitally stored. The following variables were considered longitudinally: vital capacity (VC), total lung capacity (TLC), FEV_1_, FEV_1_/VC-ratio, and the diffusion capacity using the single-breath method (DLCO). This study focused only on FEV_1_ and cDLCO. Because LFS is composed of percentage of predicted values of cDLCO and FEV_1_, we also used percentages of predicted values for better comparability. Predicted values were calculated according to Cotes et al and Quanjer et al (24,25) and DLCO was adjusted to the hemoglobin level (cDLCO).

### Statistical analysis

All statistical analyses were performed using SPSS, 23.0 (IBM, Corporation, Armonk, NY, USA). Χ^2^ test was used to compare two categorical variables and analysis of variance was used to compare multiple categorical variables. Brown-Forsythe test was used if homoscedasticity was not assumed. Post-hoc analysis was done with the Scheffé procedure or, in case of unequal distribution of variances, with Dunnett-T3 test. For description of the time course of pulmonary function parameters matched-pair analysis was used. For OS, actuarial curves were obtained by the Kaplan-Meier analysis and compared using the log-rank test. To assess the relation between LFS, cDLCO, and FEV_1_ and the development of cGvHD, Cox-regression analysis was used. Stem cell source, GvHD prophylaxis, acute GvHD, related or unrelated donor, female donor into male recipient, reduced intensity or myeloablative conditioning, busulfan in the conditioning regimen, ECOG before HSCT, CMV-reactivation risk, thoracic radiation, total body irradiation, history of smoking, age over 40 years, and T-cell depletion were tested in a forward and backward analysis as covariates. As acute GvHD (none or grade 1 vs grade 2-4: hazard ratio [HR] 1.855 (1.204-2.857), *P* = 0.005) and reduced intensity vs myeloablative conditioning (HR 1.584 [1.027-2.441], *P* = 0.037) had significant influence on the development of cGvHD, these covariates were included in the final analysis. In all analyses a two-sided significance level of α = 0.050 was considered significant.

## Results

### Time course of FEV_1_, cDLCO, and LFS after allo-HSCT

**Figure 1:**
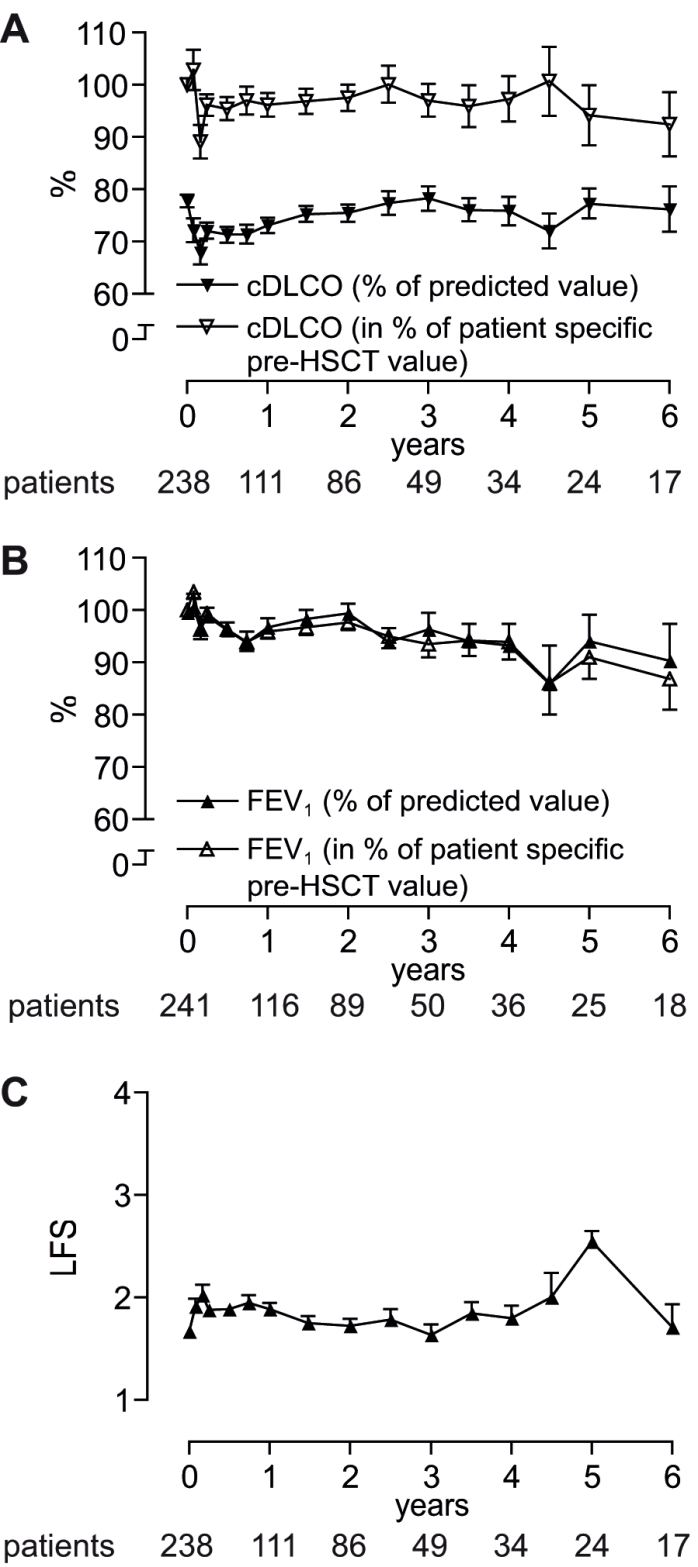
Time course of diffusion capacity of the lung for carbon monoxide corrected for hemoglobin level (cDLCO) (**A**), forced expiratory volume in the first second (FEV_1_) (**B**) and lung function score (LFS) (**C**). Values represent the mean of pulmonary function testing (PFT) result of all patients measured at a given time point. For FEV_1_ and cDLCO, both percentage of predicted values (solid triangle) and percentage of patient-specific pre-allo-hematopoietic stem cell transplantation (HSCT) value (hollow triangle) are shown. Percentages of predicted values (solid triangle) were used to calculate LFS. Tables below indicate the number of patients for whom PFT was performed at a given time point.

Changes over time of cDLCO, FEV_1,_ and LFS in surviving patients are demonstrated in Figure 1. Pre-allo HSCT cDLCO values significantly correlated with cDLCO up to 4 years after allo-HSCT, pre-allo HSCT FEV_1_ values significantly correlated with FEV_1_ up to 6 years after allo-HSCT, and pre-HSCT LFS significantly correlated with LFS values up to 4 years after allo-HSCT (data not shown).

**Figure 2:**
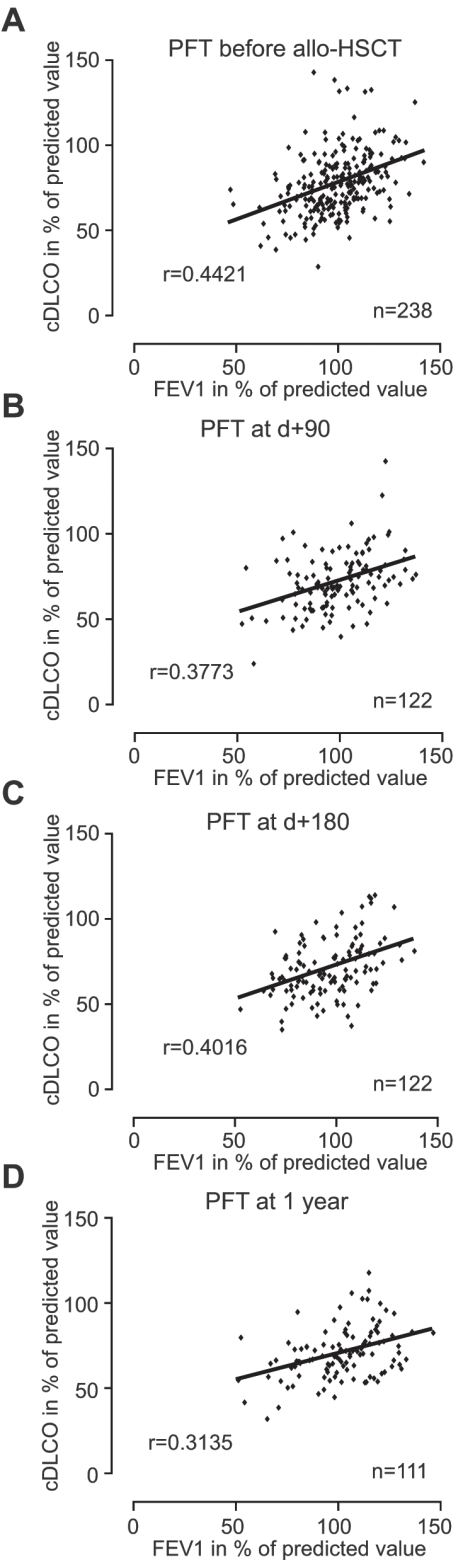
Linear regression curves of forced expiratory volume in the first second (FEV_1_) and carbon monoxide corrected for hemoglobin level (cDLCO) in percentages of predicted value before allo-hematopoietic stem cell transplantation (HSCT) (**A**), *P* < 0.001), and 3 (**B**), *P* < 0.001), 6 (**C**), *P* < 0.001), and 12 months (**D**), *P* < 0.001) after it.

cDLCO and FEV_1_ showed a weak but significant positive correlation before allo-HSCT (r = 0.4421; Figure 2A), at 3 (r = 0.3773; Figure 2B), at 6 (r = 0.4016; Figure 2C) and at 12 months after allo-HSCT (r = 0.3135; Figure 2D), and changes in cDLCO more than those in FEV_1_ contributed to an increase in LFS.

### Influence of pre- and post-transplantation pulmonary function on overall survival

**Figure 3:**
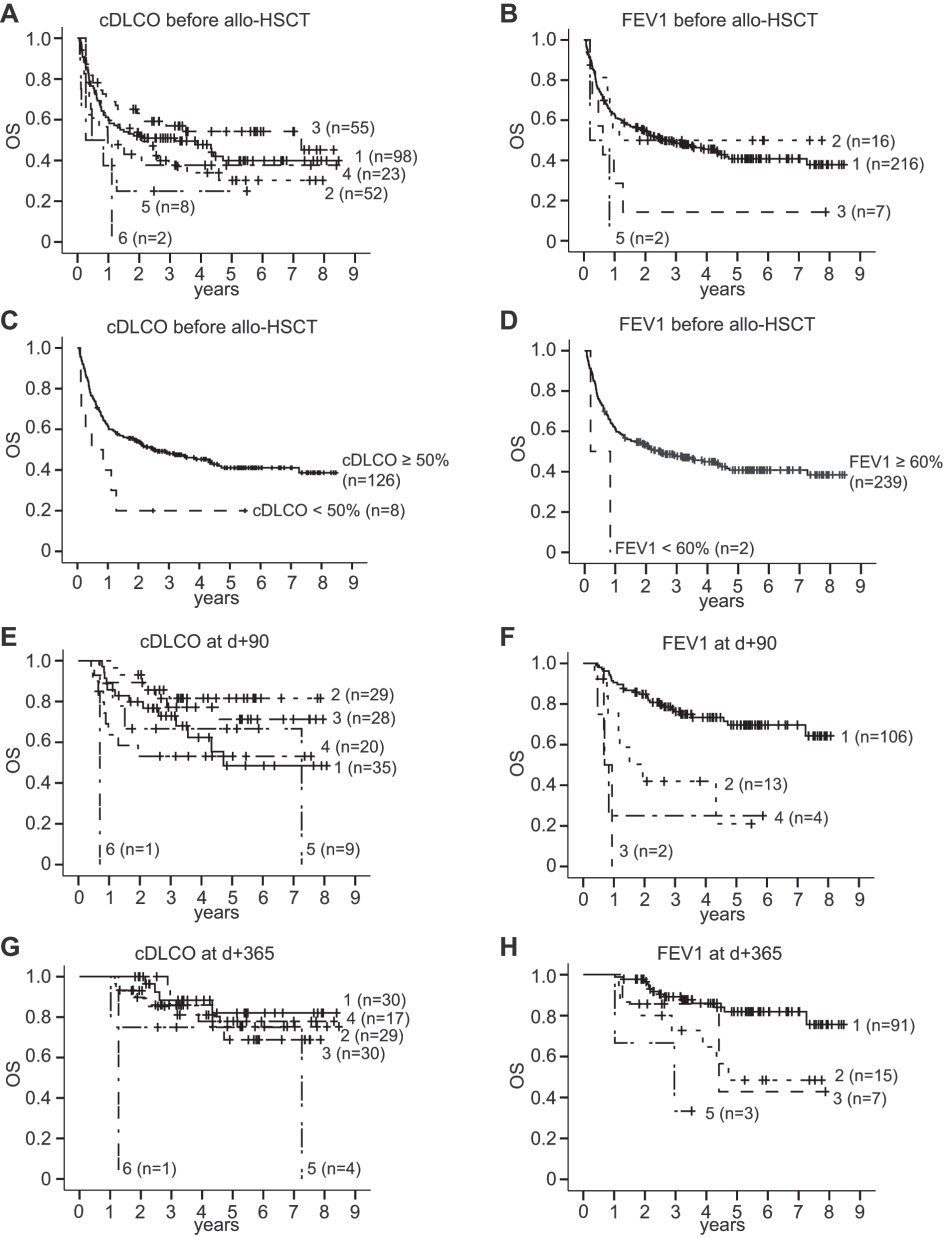
Overall survival in relation to diffusion capacitiy for carbon monoxide corrected for hemoglobin level (cDLCO) and forced expiratory volume in the first second (FEV_1_) values before allogeneic hematopoietic stem cell transplantation (allo-HSCT) (**A-D**), and at 3 and 12 months (**E-H**) after allo-HSCT. Pulmonary function testing (PFT) intervals for cDLCO and FEV_1_ are grouped as follows: 1: ≥80%; 2: 70%-80%; 3: 60%-70%; 4: 50%-60%; 5: <50% of predictive value in panel (**A**), (**B**), and **E-H**; in panel **C,** cDLCO is divided in ≥50% vs <50%; and in panel (**D**), FEV_1_ is grouped in ≥60% vs <60% of predicted value. Linear *P* values obtained using log rank test are: panel (**A**) (*P* = 0.351), panel (**B**) (*P* = 0.025), panel (**E**) (*P* = 0.187), panel (**G**) (*P* = 0.090), panel (**C**) (*P* = 0.052), panel (**D**) (*P* = 0.040), panel (**F**) (*P* < 0.001), and (**H**) (*P* < 0.001).

We next determined the influence of pre-transplantation PFT parameters on clinical outcome. Pre-HSCT cDLCO showed no linear relation with OS (Figure 3A). Yet, patients with cDLCO<50% of predicted value had significantly lower OS than patients with cDLCO≥50% (20.0% vs 41.1%, *P* = 0.025, Figure 3C). After we classified pre-HSCT FEV_1_ values by 10% increments, a trend but not a significant impact of decreased FEV_1_ on OS was observed (*P* = 0.052, Figure 3B). However, patients with pre-HSCT FEV_1_<60% of predicted value had significantly shorter OS than patients with pre-HSCT FEV_1_≥60% (0% vs 38.4%, *P* = 0.040, Figure 3D).

After allo-HSCT, no relation between OS and cDLCO was seen at 3 (*P* = 0.187; Figure 3E) and 12 months (*P* = 0.090; Figure 3G). In contrast, decreased FEV_1_ demonstrated a significant relation with OS at both time points (both *P* < 0.001, Figure 3F+H).

**Figure 4:**
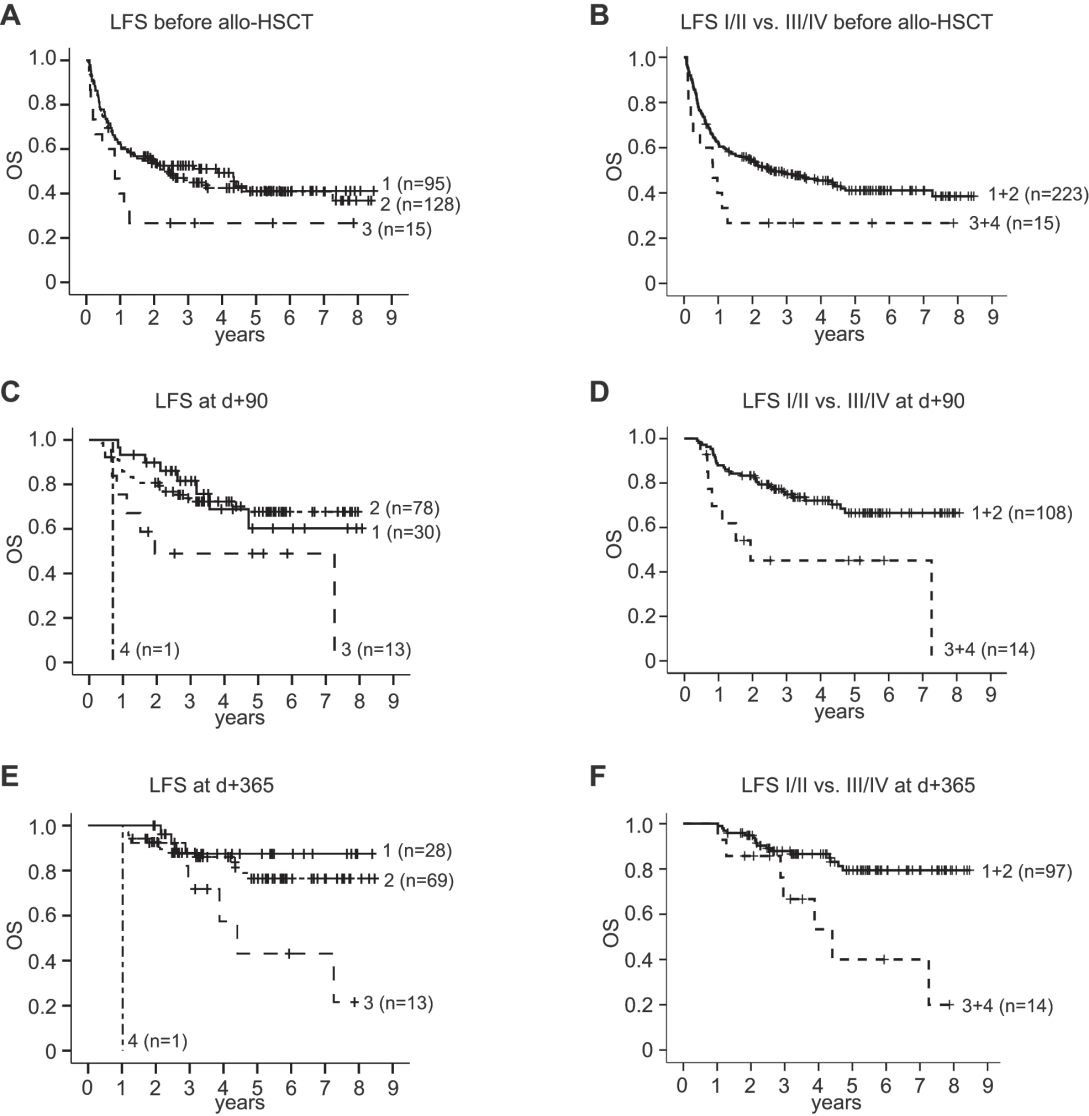
Overall survival in relation to lung function score (LFS) values before allogeneic hematopoietic stem cell transplantation (allo-HSCT) (**A, B**), at 3 (**C, D**) and at 12 months (**E, F**) after allo-HSCT. LFS grades I-IV as defined in Table 1 (**A, C, E**) and grouped into I and II vs III and IV in **B, D** and (**F**). Linear *P* values obtained using log rank test are panel (**A**) (*P* = 0.191), panel (**B**) (*P* = 0.069), panel (**C**) (*P* = 0.028), panel (**E**) (*P* = 0.002), panel (**D**) (*P* = 0.005), and panel (**F**) (*P* = 0.001).

Although no significant relation was found between pre-HSCT LFS and OS, shorter OS was observed with an increase in LFS grade, but the trend was not significant (5-year OS LFS I: 41.2%; LSF II: 36.8%; LFS III: 26.7%, *P* = 0.191, Figure 4A), suggesting LFS≥III can be considered a predictive threshold of shorter survival. Patients with a pre-HSCT LFS III/IV had a shorter overall survival than patients with pre-HSCT LFS I/II (307 vs 918 days respectively, *P* = 0.069, Figure 4B). OS was significantly shorter in patients with a baseline LFS III compared to patients with LFS I (median OS 307 vs 2208 days, *P* = 0.037, not shown).

After HSCT, increased LFS showed strong influence on OS at both 3 (*P* = 0.028; Figure 4C) and 12 months (*P* = 0.002; Figure 4E), confirming LFS III/IV as a critical threshold at either time point (3 months: *P* = 0.005; Figure 4D; 12 months: *P* = 0.001; Figure 4F).

### Relationship between cGvHD and LFS

LFS has been proposed as a parameter in the assessment of chronic pulmonary GvHD (20). Therefore, we tested whether LFS values predicted the occurrence of cGvHD in our patient cohort. Of the 241 patients, 109 (45%) developed cGvHD, 14.7% until day +120, 25% until day +142, 50% until day +180, 75% until day +229, and 87% after one year (median time of onset: 180 days, range 94-1912 days). As mentioned above, acute GvHD (none or grade 1 vs grade 2-4: HR 1.855 (1.204-2.857), *P* = 0.005) and reduced intensity vs myeloablative conditioning (HR 1.584 [1.027-2.441], *P* = 0.037) had significant influence on the development of cGvHD in the unadjusted model, resulting in the inclusion of these covariates in the final analysis.

Three months after HSCT, we evaluated cDLCO in 122 patients and FEV_1_ in 125 patients. Out of these, 69 and 72 patients, respectively, developed cGvHD. Neither in the adjusted nor in the unadjusted Cox-regression model LFS, cDLCO, and FEV_1_ were related to the development of cGvHD (not shown).

Six months after allo-HSCT, we evaluated cDLCO in 122 patients and FEV_1_ in 126 patients. Out of these, 74 and 75 patients, respectively, developed cGvHD. Unadjusted Cox-regression model showed that decreased FEV_1_ was significantly related to the development of cGvHD (*P* = 0.030, not shown), while changes in cDLCO and LFS were not. After adjustment for acute GvHD and conditioning regimen (reduced intensity vs myeloablative), there was still a relation between decreased FEV_1_ and development of cGvHD but it was not significant anymore (*P* = 0.069, Table 3), which might be due to very small number of patients with decreased FEV_1_.

**Table 3:** Cox-regression analysis for the risk of developing chronic graft vs host disease (cGvHD) depending on pulmonary function parameters at day +180 and day +365. The adjusted model included acute GvHD and reduced intensity vs myeloablative conditioning as covariates*

	Day +180			Day +365		
Parameter	N	adjusted HR (95% CI)	*P*	N	adjusted HR (95% CI)	*P*
LFS grade						
I	33	referent		28	referent	
II	70	0.779 (0.457-1.330)	0.360	69	0.970 (0.546-1.721)	0.916
III	19	0.788 (0.370-1.677)	0.536	13	3.313 (1.547-7.095)	0.002
IV	0			1	3.540 (0.451-27.804)	0.229
		trend	0.640		trend	0.002
FEV_1_						
>80%	103	referent		91	referent	
70%-80%	15	0.852 (0.407-1.784)	0.671	15	1.418 (0.719-2.796)	0.313
60%-70%	6	1.419 (0.432-4.661)	0.564	7	3.029 (1.170-7.844)	0.022
50%-60%	1	27.423 (2.783-270.184)	0.005	0		
40%-50%	1	0 (0-8.66x10^213)	0.970	3	2.605 (0.775-8.761)	0.122
<40%	0			0		
		trend	0.069		trend	0.054
cDLCO						
>80%	34	referent		30	referent	
70%-80%	24	0.701 (0.349-1.407)	0.317	29	0.885 (0.453-1.728)	0.720
60%-70%	34	0.742 (0.406-1.356)	0.333	30	0.963 (0.497-1.867)	0.911
50%-60%	20	0.545 (0.249-1.194)	0.129	17	1.568 (0.722-3.403)	0.256
40%-50%	8	1.346 (0.507-3.576)	0.551	4	2.867 (0.952-8.629)	0.061
<40%	2	1.604 (0.373-6.901)	0.525	1	3.000 (0.382-23.581)	0.296
		trend	0.439		trend	0.241

*HR – hazard ratio; LFS – lung function score; FEV_1_ – forced expiratory volume in the first second; cDLCO –.diffusion capacity of the lung for carbon monoxide corrected for hemoglobin level.

One year after allo-HSCT, cDLCO was evaluated in 111 and FEV_1_ in 116 patients, out of these 71 and 72, respectively, developed cGvHD. Both adjusted and unadjusted Cox-regression model showed a significant influence of LFS on the development of cGvHD (Table 3, *P* = 0.002 for both). In the unadjusted model decreased FEV_1_ showed a trend toward a relation with the occurrence of cGvHD (*P* = 0.107, not shown), which became almost significant in the adjusted model (Table 3; *P* = 0.054). There was no relation between cDLCO and cGVHD development.

Eighteen months after allo-HSCT, cDLCO was evaluated in 104 and FEV_1_ in 106 patients. Out of these, 67 patients developed cGvHD at the time of PFT or subsequently. Only FEV_1_ showed a significant influence on cGvHD in the unadjusted and adjusted model (not shown, *P* = 0.036 and 0.009. respectively).

### Decrease in FEV_1_ after day +90 and incidence of cGvHD

We further determined the difference in FEV_1_ at day +180 and day +365 compared to day +90, as well as at day +180 compared to day +365, and considered a 10% decrease as relevant. A decrease of more than 10% from day +90 to day +180 was seen in 19 out of 83 patients in whom PFT was done; from day +90 to day +365 in 23 of 87 patients; and from +180 and day +365 in 16 of 89 patients.

The incidence of treatment related mortality did not significantly differ between the patients with a relevant FEV_1_ decline and the patients with stable or increased FEV_1_ between day +90 and day +180 as well as between day +90 and day +365, but it increased in patients with a FEV_1_ decline between day +180 to day +365 from 5.5 to 25% (Table 4). In addition, 15 patients with a decrease in FEV_1_ between day +180 and day +365 had a higher incidence of cGvHD (93.8 vs 53.4%), pulmonary GvHD (56.3 vs 11.0%), and a lower OS (56.3 vs 82.2%) than 39 patients with stable FEV_1_ in this period. In contrast, OS did not differ between patients with or without FEV_1_ decline between day +90 and day +180 or day +90 and day +365. The incidence of pulmonary cGvHD did not differ in patients with or without FEV_1_ decline between day +90 and day +180, whereas the incidence of lung disease in patients with a decline of FEV_1_>10% between day +90 and day +365 was 26.1% compared to 9.4% only in patients with stable FEV_1_. The incidence of cGvHD irrespective of specific organ manifestations did not significantly differ (47.4% vs 59.5% day +90 until +180; 65.2% vs 56.3% day +90 until day +365) between patients with a FEV_1_ decline and those with stable FEV_1_ (Table 4).

**Table 4:** Incidence of chronic graft vs host disease (cGvHD) overall, cGvHD of the lung, treatment related mortality (TRM), and overall survival (OS) in patients with a decline of FEV_1_ from day +90 to day +180 or to day +365, or from day +180 to day +365, of more than 10% compared to patients with no change or a decrease of less than 10%

Time period	FEV_1_ decline >10%	cGvHD % (n)	cGvHD lung % (n)	TRM % (n)	OS % (n)
Day +90 – day +180	no	59.5 (44)	16.2 (12)	12.2 (9)	64.9 (48)
	yes	47.4 (9)	15.8 (3)	10.5 (2)	73.7 (14)
Day +90 – day +365	no	56.3 (36)	9.4 (6)	6.3 (4)	84.4 (54)
	yes	65.2 (15)	26.1 (6)	8.7 (2)	78.3 (18)
Day +180 – day +365	no	53.4 (39)	11.0 (8)	5.5 (1)	82.2 (60)
	yes	93.8 (15)	56.3 (9)	25.0 (4)	56.3 (9)

## Discussion

A promising approach to improve the understanding and treatment of cGvHD was the NIH Consensus Development Project on criteria for clinical trials in cGvHD. One goal of this project was to improve the clinical assessment of pulmonary cGvHD by proposing LFS as a grading score for pulmonary cGvHD (20). In the new diagnostic and response criteria of the National Institutes of Health Consensus Development Project, the lung function score (26,27) is no longer recommended and FEV_1_ as single parameter to assess GvHD of the lung is suggested (27), which confirms our finding that cDLCO has no relation to the development of cGvHD.

In our study, overall survival was related to FEV_1_ and LFS. Pre-HSCT FEV_1_ showed a higher influence on overall survival than LFS and cDLCO. FEV_1_<60% and cDLCO<50% were associated with inferior survival, consistent with prior reports (28). Combining pre-HSCT cDLCO with FEV_1_ may translate into better ability to identify groups at increased risk for treatment-related mortality, but this is not supported by our data.

Parimon et al (19) demonstrated a stronger relation between OS and a differently defined pre-HSCT LFS in a very large patient cohort. This discrepancy might be explained by the smaller number of patients in our study and differences in LFS categorization (in the study by Parimon et al FEV_1_ and cDLCO where scored with 1 for >80%, 2 for 70%-80%, 3 for 60%-70%, and 4 for <60%, composed in a LFS grade of I for 2, II for 3-4, III for 5-6 and IV for 7-8 points).

After allo-HSCT, both decreased FEV_1_ and increased LFS levels were associated with shorter OS, suggesting that both FEV_1_ and LFS are useful parameters in assessing the impact of pulmonary function loss after allo-HSCT on clinical outcome. Again, while it seems reasonable to hypothesize that the LFS has a higher clinical value compared to the use of FEV_1_ alone and this might result from combining the LFS constituting compounds FEV_1_ and cDLCO, this was not shown in our study. According to the current guidelines of the ATS/ERS taskforce (29), FEV_1_ can be used to measure the severity of obstructive and restrictive changes in pulmonary function, as either corresponds to a decrease in FEV_1_. Pulmonary damage due to different patterns of pulmonary disease will be merged together within the LFS: Airflow obstruction is a common complication after allo-HSCT (30,31), and in some cases evolves from/to BO (32-34); restrictive changes, accompanied by a reduced FEV_1_, have been frequently reported (10,31,32,35-37); and a reduced cDLCO has been observed in many patients already prior to allo-HSCT, often followed by a temporary decline and by a partial recovery after transplantation (28,35,38). In addition, decreased cDLCO is found in numerous pulmonary complications following allo-HSCT, not only including late onset non-infectious lung injury, but also early complications such as clinical or subclinical alveolitis and interstitial pneumonitis, pulmonary hemorrhage, engraftment syndrome or pulmonary vascular disease, and presents as reversible pulmonary toxicity secondary to conditioning regimens (4,7,29,36,39-41).

Consistent with the study by Walter et al (42), we found a significant association of FEV_1_ with cGvHD at 6 and 18 months and a strong trend at 12 months after allo-HSCT. Furthermore, the incidence of cGvHD was associated with a decrease of more than 10% FEV_1_ at day +365, especially between day +180 and day +365 and resulted in elevated treatment-related mortality and reduced survival. One year after allo-HSCT we also showed a significant relation of LFS with cGvHD. We also showed that cDLCO<50% potentially contributed to the LFS interrelation with cGvHD, but it alone was not related to cGvHD.

In contrast to our study, which showed no significant association between impaired FEV_1_, cDLCO, and LFS values at day +90 and overall development of cGvHD, Walter et al (42) demonstrated a significant association of high LFS at day +80 with the development of cGvHD within one year after HSCT, attributing their observation mostly to a decrease in FEV_1_ rather than cDLCO. The different results may be explained by a different composition of the patient-specific LFS values, as in our cohort only 5% of patients had a FEV_1_ below 70% compared to 11% in the study by Walter et al. Also, a transitional decrease in lung function determined by PFT can occur in this time period post HSCT due to non-GvHD causes (38,39,43). The relatively early drop in pulmonary function, mainly reflected by a decrease in cDLCO, might be attributed to infectious complication or cytokine-mediated effects after allo-HSCT (4,7,44,45). Walter et al further restricted their data to patients developing cGvHD within one year after HSCT, whereas in our study no such time limit was set. Patients developing cGvHD at later time points can have normal LFS at day +90, therefore showing no relation between day +90 LFS and cGvHD, as observed in our cohort. Furthermore our study population is smaller than the one evaluated by Walter et al (42), therefore our study is potentially underpowered to detect a (minor) predictive role of LFS at 3 months for survival and for Cox-regression models with up to 5 different categories as assumed by inconsistent hazard ratios for FEV_1_ at 12 months as well as for FEV_1_, cDLCO, and LFS at 3 months.

Another limitation of our study was that since only patients transplanted until 2005 were included in the analysis, severity grading of cGVHD was not performed according to the NIH consensus (27,46). Conditioning regimens as well as GvHD prophylaxis and treatment approaches may differ between centers, therefore possibly limiting the results of our study. However, up to now calcineurin inhibitor plus methotrexate have remained the gold standard and response rates for second line treatment in steroid refractory GvHD rates are similar across different approaches and no definite recommendation as to which is superior can be given.

Also, we compared the lung function with overall cGvHD rather than with lung GvHD. In our cohort of 241 patients, only 24 had symptomatic lung GvHD, therefore statistical analysis has to be interpreted with caution due to small patient number. During the follow-up, FEV_1_ decreased slightly, which might be due to long-term toxicity, but also due to mild cGvHD not clinically affecting the lungs or cGVHD resulting in subtle changes within the lung.

This study showed that FEV_1_ as a single parameter had a strong association with both OS and cGvHD at most time points before and after allo-HSCT. However cDLCO did not show such an association, which gives only limited support for the application of the LFS as defined by the NIH Consensus Project on cGvHD (20) with respect to its predictive value on transplantation outcome and its relation with cGvHD. Therefore, prospective trials investigating the value of LFS combining FEV_1_ and cDLCO as a predictor of treatment response are needed. The presented results further allow to formulate clinically relevant implications, such as a) a regular screening of FEV_1_ after allo-HSCT identifies patients with lung manifestations of cGvHD, while cDLCO appears to be only of clinical relevance if <50% of the predicted normal value, b) the assessment of FEV_1_ at day +90 is recommended as baseline to assess the toxicity of the conditioning regimen, but is unlikely to detect changes already related to pulmonary cGvHD, c) the majority of patients developing pulmonary cGvHD show a decline of FEV_1_ between day +180 and day +365 after allo-HSCT and d) reduction of FEV_1_>10% compared to baseline is associated with increased morbidity and mortality. Additionally, novel parameters like acinar airways ventilation heterogeneity and lung clearance index (47) might evolve as markers for early diagnosis of pulmonary involvement in cGvHD, and their evaluation alone or in combination with LFS or FEV_1_ is warranted.
